# Correction: Zossou et al. Radiomics-Based Classification of Tumor and Healthy Liver on Computed Tomography Images. *Cancers* 2024, *16*, 1158

**DOI:** 10.3390/cancers17061021

**Published:** 2025-03-18

**Authors:** Vincent-Béni Sèna Zossou, Freddy Houéhanou Rodrigue Gnangnon, Olivier Biaou, Florent de Vathaire, Rodrigue S. Allodji, Eugène C. Ezin

**Affiliations:** 1 Université Paris-Saclay, UVSQ, Univ. Paris-Sud, CESP, Équipe Radiation Epidemiology, 94805 Villejuif, Francerodrigue.allodji@gustaveroussy.fr (R.S.A.); 2 Department of Clinical Research, Radiation Epidemiology Team, Gustave Roussy, 94805 Villejuif, France; 3 Centre de Recherche en Épidémiologie et Santé des Populations (CESP), U1018, Institut National de la Santé et de la Recherche Médicale, 94805 Villejuif, France; 4 Ecole Doctorale Sciences de l’Ingénieur, Université d’Abomey-Calavi, Abomey-Calavi 384, Benin; 5 Department of Visceral Surgery, CNHU-HKM, Cotonou 229, Benin; 6 Department of Radiology, CNHU-HKM, Cotonou 229, Benin; 7 Institut de Formation et de Recherche en Informatique, Université d’Abomey-Calavi, Abomey-Calavi 384, Benin; eugene.ezin@uac.bj; 8 Institut de Mathématiques et de Sciences Physiques, Université d’Abomey-Calavi, Dangbo 384, Benin

**Text Correction**

There was an error in the original publication [1]. The term “benign tumors” was used to refer to “liver metastases”, and the term “malignant tumors” was used for “HCC”.

Corrections have been made to the following sections of the manuscript: Simple Summary; Section 1. Introduction, paragraph 5; Section 2. Materials and Methods, Subsection 2.1. Proposed System, paragraph 1; Section 3. Results, Subsection 3.4. Construction and Evaluation of the Classification Model of HCC and Metastatic Tumors; Section 4. Discussion, paragraph 1 and paragraph 3; Section 5. Conclusions. The corrected paragraphs are shown below.

**Simple Summary**: Liver malignancies, particularly hepatocellular carcinoma, and metastases stand as prominent contributors to cancer mortality. Within abdominal computed tomography imaging, much of the data remain underused by radiologists. Radiomics uses advanced image analysis to extract quantitative features from medical scans for deeper diagnosis, treatment, and prognosis insights. Machine learning algorithms enable analyzing these features, facilitating an automatic, rapid, and efficient medical management process. We used these algorithms to train models that can distinguish between healthy livers and those with tumors, as well as between HCC and metastatic tumors, using CT images from the electronic medical records of the Centre National Hospitalier Universitaire Hubert Koutoukou Maga (CNHU-HKM) in Benin. The high correlation scores suggest that the radiomics signature is a prognostic biomarker for hepatic tumor screening.

**1. Introduction**

Recent research has demonstrated significant potential in using radiomics to identify variables associated with clinical outcomes [17,18]. However, only a few studies have used the entire liver as the region of interest (ROI) in their research. Additionally, no African investigations have been conducted on applying radiomics or machine learning to classify liver lesions. This study evaluates the effectiveness of radiomic features extracted from CT scans of the liver in distinguishing between healthy and cancerous tissues and differentiating HCC from metastatic tumors, thereby leveraging radiomics for nuanced diagnostic insights into liver health. To our knowledge, this is the first study in Africa.

**2. Materials and Methods**

*2.1. Proposed System*

A machine learning (ML) model has been developed to accurately classify healthy livers and livers with tumors using radiomic features extracted from CT images. There are five stages in the workflow process of the proposed system: segmentation of ROIs, feature extraction, statistical analysis, feature selection, and classification. The entire liver was interactively evaluated as the ROI. Figure 1 shows the block diagram of the proposed system. The work consists of two main phases: classifying healthy and tumor livers. Then, among those with tumors, classifying them as HCC or metastases.

**3. Results**

*3.4. Construction and Evaluation of the Classification Model of HCC and Metastatic Tumors*

**4. Discussion**

This study shows that machine learning algorithms can capture the difference between normal and tumor liver tissue and can detect HCC and metastatic tumors in CT images at the portal phase using radiomic features. In radiomics, a significant array of features, often numbering in the hundreds, is generated to delineate a specified ROI through various methodologies [29]. These features undergo evaluation for their potential as prognostic indicators. Additionally, the critical process of feature selection demands a focus on their consistency and responsiveness concerning the delineation methodology for them to be deemed clinically viable. To date, no studies have systematically assessed the application of various machine learning algorithms in differentiating between tumor and non-tumor tissues using the entire liver as the ROI. Furthermore, no recorded African studies have employed machine learning to classify liver lesions.

In contrast, only naive Bayes and LASSO regression yielded satisfactory scores for detecting HCC and metastatic tumors. The other models produced low scores. In total, 1686 features were extracted from portal-phase CT images. Twenty-two features showed a higher correlation in the presence of tumor or no tumor classification, while there were eighteen in the classification of HCC and metastatic tumors.

**5. Conclusions**

This research conducted a preliminary radiomics investigation using CT scans. A correlation was established between radiomic features and the distinction between healthy and tumor liver tissue, on the one hand, and between HCC and metastatic tumors, on the other hand. The naive Bayes model shows great results for the two tasks. It achieved an AUROC of 0.9268 (95%CI: 0.8224–1) in the distinction between healthy and tumor liver tissue and an AUROC of 0.8571 (95%CI: 0.6764–1) in the distinction between HCC and metastatic tumors. This result demonstrates that the developed radiomics signature is statistically significantly correlated with healthy liver tissue and liver tumor tissue using the entire liver as a ROI. However, there is a need for larger retrospective and prospective research on liver CT scans that examines potential prognostic markers and has strict reference criteria.

**Figure/Table Legends**

In the original publication [1], there was a mistake in the titles of Table 7, Table 8, and Figure 6. The term “benign tumors” was used to refer to “liver metastases” and the term “malignant tumors” was used for “HCC”. The correct titles appear below.

**Table 7.** Metric measures for each ML algorithm in HCC and metastatic tumor classification.

**Table 8.** The most important radiomic features in HCC and metastatic tumor classification.

**Figure 6.** The ROC curves of the classification model of HCC and metastatic tumors.

**Missing Citation**

In the original publication [1], “Candita, G.; Rossi, S.; Cwiklinska, K.; Fanni, S.C.; Cioni, D.; Lencioni, R.; Neri, E. Imaging Diagnosis of Hepatocellular Carcinoma: A State-of-the-Art Review. *Diagnostics* **2023**, *13*, 625” was not cited. The citation has now been inserted in Introduction, paragraph 3, and should read as follows:

The approach to liver cancer treatment varies based on the tumor’s phenotype [5,6]. Initially, radiologists employed MRI or CT scans to assess the phenotype, and various manual grading criteria for liver tumors have been established [7]. However, the visual characteristics of these lesions on scans can greatly differ due to histological variations [8], leading to subjective interpretations and a lack of consensus on their exact definitions [7]. While tissue biopsy offers definitive histological confirmation, it is not routinely required for HCC diagnosis thanks to advancements in non-invasive methods utilizing characteristic imaging features and radiomics [9].

The authors apologize for any inconvenience caused and state that the scientific conclusions are unaffected. This correction was approved by the Academic Editor. The original publication has also been updated.
